# Trophoblast Organoids: Capturing the Complexity of Early Placental Development In Vitro

**DOI:** 10.3390/organoids3030012

**Published:** 2024-08-02

**Authors:** Brady M. Wessel, Jenna N. Castro, Victoria H. J. Roberts

**Affiliations:** Division of Reproductive and Developmental Science, Oregon National Primate Research Center (ONPRC), Oregon Health and Science University (OHSU), Beaverton, OR, 97006, USA

**Keywords:** first trimester placenta, in vitro model, trophoblast organoids, trophoblast, differentiation, nonhuman primate

## Abstract

First trimester placental development comprises some of the most critical yet understudied events that impact fetal development. Improper placentation leads to a host of health issues that not only impact the fetal period but also influence offspring throughout their lives. Thus, a paradigm to study early placental development is necessary, and this has spurred on the pursuit of new in vitro model systems that recapitulate specific aspects of placentation. One of the most complex and translationally valid models to arise are organoids, three-dimensional structures comprising multiple differentiated cell types that originate from a common progenitor population. Trophoblasts are the progenitor cells of the placenta, serving as the proliferative base for placental development. Recent advances have enabled the derivation of organoids from primary tissue, yet access to first trimester human samples is ethically constrained; derivation from established trophoblast stem cell lines is an alternative source. Organoids have already proven useful in generating insights into molecular events that underlie trophoblast differentiation, with the identification of new cell subtypes that are primed to differentiate down different paths. In this review, (1) we recap early pregnancy development events, (2) provide an overview of the cellular complexity of the placenta, (3) discuss the generation of organoids from tissue versus cellular sources, (4) highlight the value of translational animal models, and (5) focus on the complexities of the molecular regulation of trophoblast organoid development, differentiation, and function.

## 1. Introduction

The placenta represents a crucial yet transient organ for the appropriate development of healthy offspring. Proper placentation is a complex waltz of regulation, tolerance, and inhibition between the maternal decidua and endometrium, and fetal trophoblast cells. Dysregulation of these events is suspected to contribute to, or outright cause, obstetric syndromes such as pre-eclampsia, fetal growth restriction, preterm labor, and placental abruption [1]. Despite its paramount importance, many events that govern early placental development remain unknown. Due to ethical and practical constraints, experimental studies on causal mechanisms for these diseases cannot readily be performed in human subjects. The first trimester is inaccessible to researchers, yet that is the timeframe of most importance as the placenta is being established. Critical events known to occur during the first trimester include spiral artery invasion and remodeling, to create large-bore vessels that slow the velocity of blood flow to ensure adequate maternal perfusion of the intervillous space [2]. It is at this time when dysregulation can lead to profound impacts on the developing fetus and place the health of both the mother and fetus in jeopardy. This has created a demand for placental models that reliably recapitulate early gestational timepoints. The purpose of this review is to discuss the cellular diversity that has been identified in human first trimester placentas, the considerations for utilizing trophoblast organoids (TOs), and the diversity of cell types that can be captured by the model. In addition, we discuss the nonhuman primate (NHP) as a suitable experimental system that avoids the constraints of human first trimester sampling, and the potential of this translational animal model for the study of placental biology and its application to human health.

## 2. Early Placental Development

Placentation occurs when the blastocyst implants into the maternal decidua, in the uterine wall, and rapid growth and cell proliferation follow. The first trimester placenta comprises three main regions: the trophoblastic shell, the floating villi, and the chorionic plate [3]. The floating villi and trophoblastic shell are established by trophoblast cells. These cells are derived from the trophectoderm layer of a blastocyst and begin their development into the placenta post-implantation [4]. Implantation occurs in the maternal decidua, which develops a supportive environment for the nascent embryo. Once established, the placenta must fulfill critical functions of gas exchange, nutrient transport, and waste disposal while meeting the ever-growing needs of the developing fetus. This requires a high surface area-to-volume ratio to facilitate adequate exchange capacity. To achieve this, the placenta is packed full of fetal villi: tree-like structures that branch out from the chorionic plate and occupy the intervillous space, at maturity creating upwards of 12 square meters of exchange surface area directly in contact with maternal blood in a full-term placenta [5,6]. Within the villi, there are floating villi and anchoring villi. Floating villi, as the name suggests, are free-floating in the intervillous space, anchored at their distal end to the chorionic plate. Anchoring villi connect the placenta to the decidua substrate, providing grounding for the growing placenta to connect to maternal tissue [4]. The villous tissue is the functional unit of the placenta and modeling it at the cellular level is critical to understanding fetal health and disease.

The growth and maturation of villous trees require that the trophoblast stem cell progenitor pool has the capacity to differentiate into three main cell types: cytotrophoblast (CTB), syncytiotrophoblast (SYN), and extravillous trophoblast (EVT) [4]. These three cell types operate as a well-drilled team, with each fulfilling a specific role to ensure adequate placental function. CTBs form the proliferative pool of cells for the villi, allowing for growth and extensive branching of villous trees [3]. They possess the capacity to differentiate into both EVT and SYN cell types. The tips of anchoring villi harbor cytotrophoblast columns, which are proliferative cells predisposed towards the EVT route of differentiation, whereas the floating and main stems of the villi harbor a slightly different CTB type, predisposed to SYN differentiation [4]. EVTs are the invasive cells of the placenta. They interact with the decidua and invade spiral arteries to accomplish vascular remodeling, and form the trophoblastic shell, anchoring the placenta to the decidua [4]. Several subtypes of EVTs exist, as will be discussed later in this review. The SYN type is responsible for facilitating all exchange and transport across the placenta between the maternal and fetal circulation. Formed by cell fusion with CTB progenitors just underneath them, the SYNs form a polarized, multinucleate, continuous cell layer that covers each villous tree. The SYN has two surfaces: the microvillous membrane, an apical maternal-facing side, and the basal membrane, the fetal-facing side. Substrate exchange is coordinated by uptake from the microvillous membrane and transport to the fetus via the basal membrane, and vice versa for waste removal [7]. The necessity for both in vivo and in vitro model systems as a paradigm to study first trimester placentation at the cellular level has led to a bevy of studies interrogating the cellular dynamics of this critical time.

### 2.1 Trophoblast Diversity in Placental Development

**Figure 1.**
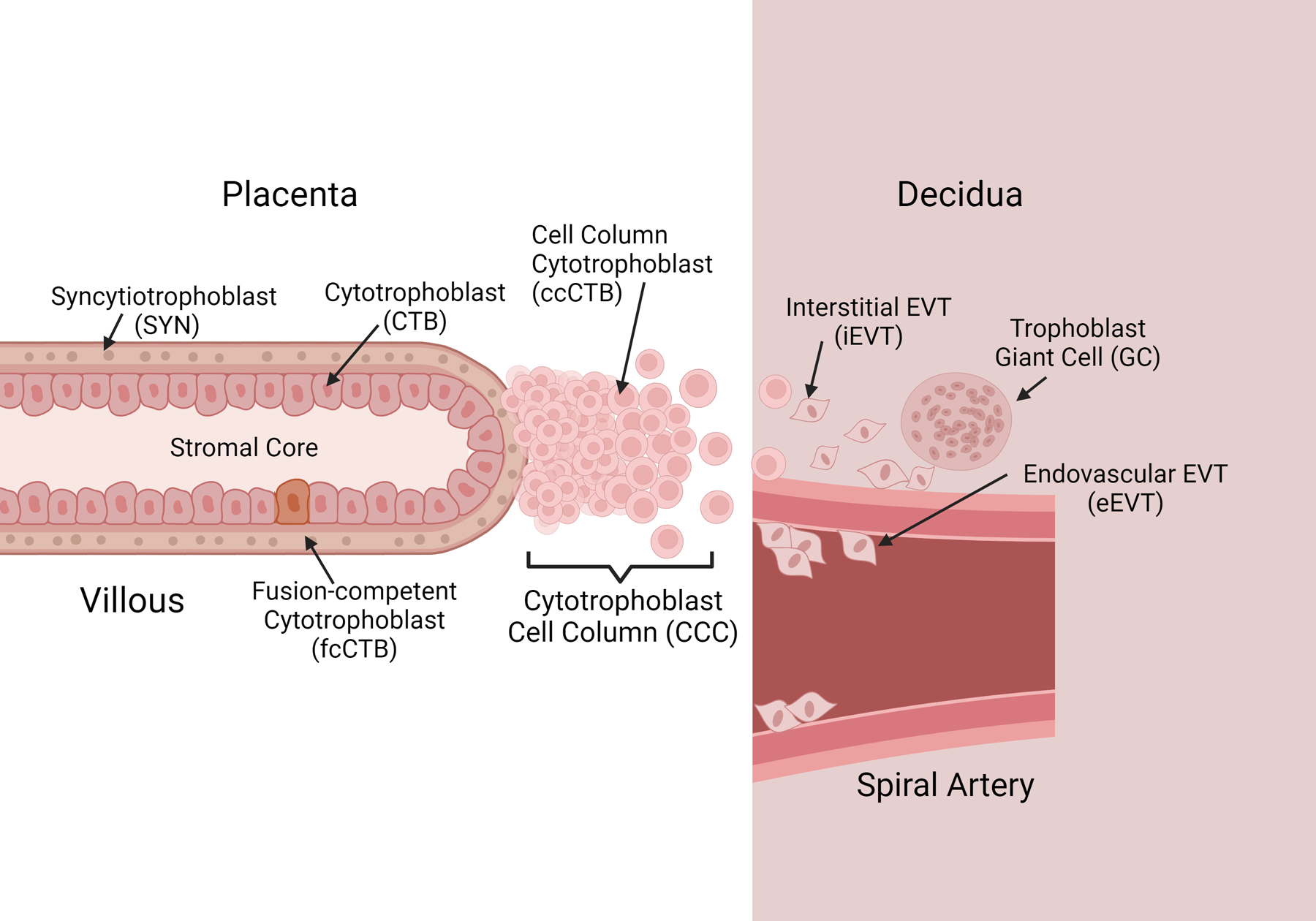
First trimester trophoblast cell phenotypes. Schematic representation of the tip of an anchoring villous at the maternal/fetal interface. The fetal side is noted in white, while maternal decidua is noted in pink. Cellular anatomy is noted with the spatial distributions of all defined trophoblast cells shown.

With researchers leveraging the power of single-cell RNA-seq, a comprehensive view of cellular dynamics in the first trimester is beginning to emerge. To discuss the TO models effectively, an understanding of key cell types in vivo is required. Indeed, while the overarching classifications of CTB, SYN, and EVT are accurate, more subtleties and differences based on both protein expression/transcriptomics as well as spatial arrangement have brought improved focus to the true diversity of these cell types. Key areas of divergence exist in the primate model, and these are also addressed. The organization and locations of these main cell types are shown in Figure 1, while Table 1 lists markers associated with each cell type.

First, to describe the CTB compartment, the bipotent progenitor cell has several states as it moves along either route of differentiation. In the villous environment, the CTB type remains proliferative, expressing stemness markers such as KI67, CCNA, TEAD4, and TP63 in addition to classic trophoblast marker KRT7 and epithelial marker CDH1 [8]. Proliferative CTBs and trophoblast stem cells (TSCs) do not appear to be identical [8]. In fact, the maintenance of the TSC niche in culture appears to be associated with slight changes that may impact the fidelity of the model for first trimester dynamics [8–10]. Yet, the TSC niche and proliferative CTB niche share considerable overlap, and further development of TSC culture conditions may alleviate these concerns. The precise definition of the TSC as opposed to the proliferative CTB remains to be uncovered, as, currently, there is no clear consensus on what defines the TSC niche, or, in fact, whether it exists in vivo. Those cells that remain in this compartment are critical to the growth and maintenance of the placenta, but some cells become quiescent, and begin to upregulate their expression of fusion proteins, particularly ERVFRD-1 [8,10–12]. At this point, the loss of proliferative activity combined with fusion protein expression suggests these cells are the progenitor population of the multinucleate SYN layer, or fusion-competent CTBs (fcCTBs). While these cells have not yet fused into the multinucleate SYNs, they are the population directly responsible for providing fusible cells. These cells do not secrete hormones that are the hallmark of mature SYN differentiation but are primed to enter the SYN niche [8,9]. Alternatively, cells located near the terminal end of an anchoring villous may enter the cytotrophoblast cell column (CCC) (Figure 1, Table 1). These cells are consistently Notch1 positive, suggesting that Notch signaling plays a role in this differentiation [13]. At the proximal end of the column, proliferative capacity is retained, with KI67-positive nuclei, but this fades as cells migrate to the distal end of the column and into the maternal decidua [13]. At the distal tip of the column, HLA-G-positive cells become much more prevalent, signaling the transition into true EVT cells.

A final component of the CTB clade is the cytotrophoblast shell. These cells are abundant early in gestation, and the shell decomposes through the first trimester until few, if any, of these cells remain by the second trimester [14,15]. Due to their ephemeral nature, they remain a critically understudied cellular component of the first-trimester placenta, but it is postulated that they function as a precursor to EVTs, allowing the placenta to find anchorage until true EVTs are differentiated and established [15]. Importantly, they also block extensive perfusion of the placenta by maternal blood, which is suggested to be a protective mechanism from oxidative stress [15]. Future studies of these cells are needed for more comprehensive characterization.

While not as diverse as the CTB compartment, the mature SYN compartment is marked by the expression of fusion proteins SDC1 and GCM1, and the expression of human chorionic gonadotrophin (hCG), which continues throughout pregnancy [11,12,16]. The SYN develops clear microvilli on its apical membrane, which increase the surface area available to perform the exchange of nutrients and oxygen. As pregnancy progresses, the SYN layer must replenish itself with a ready supply of fcCTBs, as parts of its own membrane slough off and are lost in the maternal circulation (Figure 1). Furthermore, the surface area of the SYN layer increases steadily in the first trimester as the villous trees grow and mature. The SYNs themselves have lost all proliferative capacity, and it has been suggested that older nuclei with condensed chromatin congregate into syncytial knots, which slowly increase in frequency until term [17,18]. Studying this cell type is difficult owing to the multinucleate, fused nature of the cells. Standard single-cell isolation techniques shear and destroy SYNs, so alternative approaches must be utilized. Single-nuclei isolation and subsequent RNA sequencing allow for the capture of mature SYN phenotypes [19]. These efforts have uncovered a maturation trajectory of the SYN throughout gestation, with cell trajectory modeling suggesting that the upregulation of FLT1 and PAPPA are critical to first-trimester maturation, with PAPPA marking fully matured SYNs in early samples [19]. In term samples, the FLT1 locus becomes inaccessible, with a marked decrease in FLT1 transcripts. The leveraging of singe-nucleus RNA-seq has produced considerable progress in our understanding of the previously unapproachable SYN.

Where single-cell sequencing methodologies have been of value is in revealing the diversity of the critical EVT niche. EVTs are distinguished from their trophoblast counterparts by robust expression of HLA-G, the integral marker for EVT identity. At the tips of the anchoring villi, cell-column CTBs (ccCTBs) are found (Figure 1). Marked by Notch1 expression as well as that of ITGA1/2, these cells are proliferative [13,16,20,21]. The retention of proliferative capacity in the CCC allows for the growth of new EVT cells as they migrate out of the CCC and into the decidua. It is important to note that ccCTBs are not positive for HLA-G, but at the distal end of the CCC, HLA-G is detected [13]. This is where the transition to true EVTs is marked. Integrin expression is critical to both EVT identity and the regulation of their invasion. In the CCC, expression of ITGA1 is highly prevalent, but this decreases towards the distal in of the column [16,22]. At this interface, an integrin switch is observed, where ITGA1 is downregulated in favor of ITGA5 [16,22]. It is known that ITGA5 regulates the invasive nature of EVT cells, leading them to be less invasive [22]. Upon entering the maternal decidua, two distinct forms of EVTs are observed: interstitial EVT (iEVT) and endovascular EVT (eEVT) cells. These are deeply invasive cell types. iEVT cells occupy the decidua, expressing ITGA5, PLAC8, and PAPPA2, and only invade into the inner third of the myometrium in healthy pregnancies. eEVTs are marked by NCAM1 and MMP2/12 secretion, and are attracted to the spiral artery, where they invade the lumen and replace the vascular endothelial cells [12]. This allows them to remodel spiral arteries into wider-bore vessels, optimal for perfusion of the placenta. Indeed, defects involving spiral artery remodeling are especially dire, leading to pregnancy complications (e.g., pre-eclampsia), posing a risk to the fetus and mother. Placental giant cells are a controversial trophoblast, and their exact origin is contested. It has been posited that they are left over from the primitive syncytium that forms prior to the villous placenta. Alternatively, they may be a result of terminal differentiation and aggregation of iEVTs, and recent evidence suggests this is the answer, as desmosomes present at cell–cell junctions in EVTs may regulate fusion [23,24]. Regardless, they are known to express PAPPA and other key growth factors such as VEGF [24]. Future studies may yet uncover the precise roles of giant cells in pregnancy.

The efforts of scRNA-seq studies have dramatically improved our understanding of trophoblast cellular dynamics in the first trimester. As this knowledge grows, the development of appropriate models that may capture the diversity observed in vivo and allow for experimental manipulations becomes key. Teasing apart the mechanisms of trophoblast differentiation requires a combination of in vivo and in vitro approaches.

### 2.2 In Vivo Models of Placental Development

Translational animal models are critical to studying the biological processes of placentation. Due to the constraints of accessibility of first trimester samples, many animal models have arisen to bridge the gap and inform our understanding of this critical time in development. The main model system is the mouse (*Mus musculus*). Accessible both physically and financially to many researchers, the mouse is highly favorable for its ability to generate gene-knockout models. These knockouts provide a wealth of information for a given gene of interest and offer starting points for human investigation. However, mice lack the appropriate placental anatomy to truly mimic that of humans, and more critically, postnatal developmental events are much more extensive than in humans, especially as they relate to the brain [25]. Thus, animal models that more closely recapitulate human gestation and first-trimester development are necessary.

The rhesus macaque (*Macaca mulatta*) offers a compromise between improved translational ability and cost. While not as widely accessible, the parallels between rhesus macaques and humans are numerous. It is worth mentioning that the cynomolgus macaque (*Macaca fascicularis*), another commonly used translational NHP model, is closely related to the rhesus macaque, and in terms of the placenta, anatomically identical, and by extension, its placenta is very similar to that of a human [26]. Here, we focus on the rhesus macaque due to our extensive familiarity with this species. The rhesus placenta is organized very similarly to that of a human, with a discoid shape and hemochorial organization [27]. An area of divergence is that rhesus macaques typically have bidiscoid placentas, whereas humans typically have monodiscoid placentas. Their gestational and placental growth rates are well characterized, allowing for accurate comparisons to be made across primate research [27]. This feature makes them appealing for studies using novel medical methods, such as MRI protocols to visualize oxygen levels in utero [28]. Critically, the fetal developmental milestones that occur in utero for humans are recapitulated in the rhesus macaque.

At a cellular level, humans and rhesus macaques are remarkably similar, displaying the same major cell types [29,30]. It must be noted that a few significant differences do exist between human and macaque pregnancies, namely the presence in macaques of a well-defined trophoblast shell, their depth of extravillous trophoblast invasion, and their secretion of hormones, such as monkey chorionic gonadotropin (mCG). Throughout gestation, the macaque displays a thicker, more defined cytotrophoblast shell at the junction of the decidua and placenta. In humans, this shell exists early in gestation, but it thins, and anchoring villi are responsible for forming the maternal/fetal interface [30,31]. The extent of EVT invasion is decreased in the macaque, with EVTs failing to reach the myometrium as they do in humans [32]. Macaque EVTs are less robustly characterized, owing to the lack of highly specific markers. In the rhesus, the locus for HLA-G (Mamu-G) is a pseudogene; the functional equivalent of HLA-G in the rhesus is Mamu-AG [33]. However, this protein does not localize solely to the extravillous trophoblast. Robust expression occurs on the apical membrane of the syncytiotrophoblast as well, with subtle changes throughout gestation. NCAM1 is not as specific in the rhesus as it is in the human, with expression in both eEVTs and the trophoblastic shell/CCC, but it is squarely in the realm of the EVT [29]. Integrins, specifically ITGA5, are a crucial component of EVT cell identity in both the human and the rhesus; they offer a potential specific marker of macaque EVT differentiation, representing a difference from the ITGA1 that characterizes EVTs in humans [22,34,35]. Identifying highly specific markers for macaque EVT subtypes will prove valuable to translational research.

The dynamics of NHP mCG vary significantly from profiles of human chorionic gonadotrophin (hCG). Crucially, the secretion of pregnancy-maintaining hormone hCG continues throughout gestation in humans, but in rhesus macaques, levels fall dramatically after just 30 days of gestation, as opposed to being maintained throughout pregnancy [36]. Thus, organoids derived from tissue later than gestational day 30 (roughly) may not secrete mCG, although the secretion of hCG is expected in their human counterparts. Cell-specific differences may also confound investigations using NHP models. Despite these differences, the value of NHPs in translational research is strengthened by recent single-cell sequencing data that show the remarkable ability of NHPs to capture similar placental landscapes to those of humans [34]. The maternal/fetal interface is well conserved in the primate, with key interactions between EVTs and immune cells being present in both species [34]. Thus, the use of NHPs allows researchers the opportunity to advance the field in more diverse ways than by the use of mice or human samples alone, offering the highest fidelity while alleviating the ethical concerns of modeling human placental dynamics in vivo.

### 2.3 In Vitro Models of Placental Development

Given that translational animal models have their limits, in vitro models are focused on capturing the unique aspects of human biology as it pertains to the study of the first trimester. Indeed, a wide array of in vitro models have been developed for the placenta, ranging from various cell lines to explant models and, more recently, stem cell and organoid models [37]. There are rich data sets that have been collected on primary cell cultures of first trimester trophoblasts, and these are a standard approach in the field. Where access is permissible and feasible, they offer an excellent tool with one limitation: they are short-lived. Indeed, these cultures do not tolerate many passages, and after several days in culture, they begin to form large SYN clusters. Once these have formed, the cells lose their proliferative ability. This also underlines the fact that trophoblast cells are predisposed towards the SYN pathway of differentiation. Other cell lines that are immortalized have been developed to overcome this limitation, but critically, many are derived from choriocarcinomas, and their direct trophoblast identity is questionable [20]. However, these lines have proven their worth time and again, and they offer valuable insights into aspects of trophoblast development; the researcher must choose wisely based on what fits their needs and what questions they are asking.

Placental explants are another approach to studying trophoblast differentiation and mechanics. Explants operate in two main time windows: short-term and long-term [38,39].

This is dictated by the fact that after about three days in culture, the SYN layer sloughs off and reforms [39–41]. At this time, the model loses some level of functionality, but this is restored after the reformation of the SYN layer [39,41]. Explants are a strong model because they are composed of all cell types present in the placenta, not just trophoblasts [42]. Interactions between the stromal cores of villi and trophoblasts undoubtedly contribute to appropriate development. Explants also exhibit a strong potential for differentiation under media manipulation or an altered oxygen environment [43]. Indeed, the biggest limitations on explants may well be the difficulty of obtaining enough samples, as is always a concern for early-placenta research, and the need to be efficient within the timeframes they allow for study. Furthermore, genetic manipulations cannot be performed in explant models, which is another limitation of their experimental utility.

As of 2018, the derivation of human TSCs had been achieved [44]. These cells are proliferative and stable through many passages. In EVT differentiation media, HLA-G-positive EVT cells appear, demonstrating the biopotency of TSCs in vitro. Crucially, when a 3D hydrogel such as Matrigel is added along with the appropriate media, TSCs can organize into 3D organoids [10]. Both primary and blastocyst-derived cells proved capable of proliferating in TSC media [44]. Dong et al., in 2020, showed that the same media cocktail can be applied to naïve induced pluripotent stem cells, yielding TSCs as well [45]. Interestingly, primed pluripotent stem cells did not express trophoblast markers under the same conditions, instead showing expression of VIM and PAX6, which is more consistent with a neuroectodermal fate. However, the need for more intensive manipulations in the lab calls into question whether these cells are faithful to trophoblast identity. Okae and company showed that their cell lines maintain genetic integrity and gene expression through high numbers of passages, which is critical for a cell culture resource.

The NHP has also seen an expansion of in vitro approaches: correlating in vivo perturbations with in vitro data is a powerful aspect of this model. The isolation of macaque trophoblast stem cells (mTSCs), reported originally in 2007 and again in 2020 by two separate groups, shows the utility of in vitro primate systems in the two most common primate model organisms, rhesus and cynomolgus macaques [35,46,47]. These cell lines have been isolated from two different starting sources. Schmidt and colleagues used primary cell isolations from first trimester placental tissue, whereas Matsumoto and company used blastocyst-derived cells and reprogrammed them into TSC-like cells [35,47]. Placenta-derived mTSCs have undergone extensive characterization to validate their identity as trophoblast cells and to demonstrate their similarity to in vivo cell types. These cells can form both SYNs and EVTs upon media-directed differentiation. Forskolin, a stimulator of cAMP production, invokes SYN characteristics, including elevated secretion of mCG compared to standard mTSCs. Interestingly, a 3D culture used for SYN spheroids showed a significant increase in mCG and progesterone, offering evidence that pursuing 3D culture models with these cells is not only possible, but may even be desirable [35]. These cells can also be directed to form EVT-like cells following the human methodology [35,44]. These EVT cells have elevated expression of Mamu-AG and NCAM1, supporting their identity. The secretion of MMP2, a crucial metalloproteinase for spiral artery remodeling, is also significantly elevated in EVT media as compared to standard mTSCs. Single-cell RNA-seq experiments also demonstrate the transcriptomic stability of these cells throughout early and late passages, which is a crucial consideration for both the stability of the line and the validity of results [35]. This provides greater accessibility to first-trimester primate samples, which is critical for translational research of humans and mechanistic studies that may inform us about mechanisms in human cells. Indeed, validation of in vitro approaches in the nonhuman primate alongside in vivo data offers a unique opportunity for mechanistic studies as well as drug screening and development.

## 3. Trophoblast Organoids as an In Vitro Model

Organoids are in vitro cultures of cells that naturally organize into structures that mimic the anatomy of an organ of interest at a cellular level. Three-dimensional culture can be achieved by suspension in extracellular matrix hydrogels or using specialized ultra-low adhesion culture plates. These conditions allow for stem cell progenitors to proliferate and spontaneously differentiate into other cell types found in the organ of interest. This complexity allows researchers to capture the complexity of cell–cell signaling in vitro. Using a chemically defined medium, changes to the system can be precisely monitored, which confers greater control over the culture system. In 2018, the first trophoblast organoid culture systems were reported [48,49]. TOs can currently be derived from primary cells, TSCs, or blastocyst cells. This allows researchers greater flexibility in deciding which route of access is most appropriate for their needs. TOs have proven capable of capturing the intricacies of in vivo first trimester placentation and offer an exciting alternative approach to dissecting trophoblast development and differentiation, which is suspected to be at the root of many pregnancy pathologies.

### 3.1 Characterization of Trophoblast Organoids

**Figure 2.**
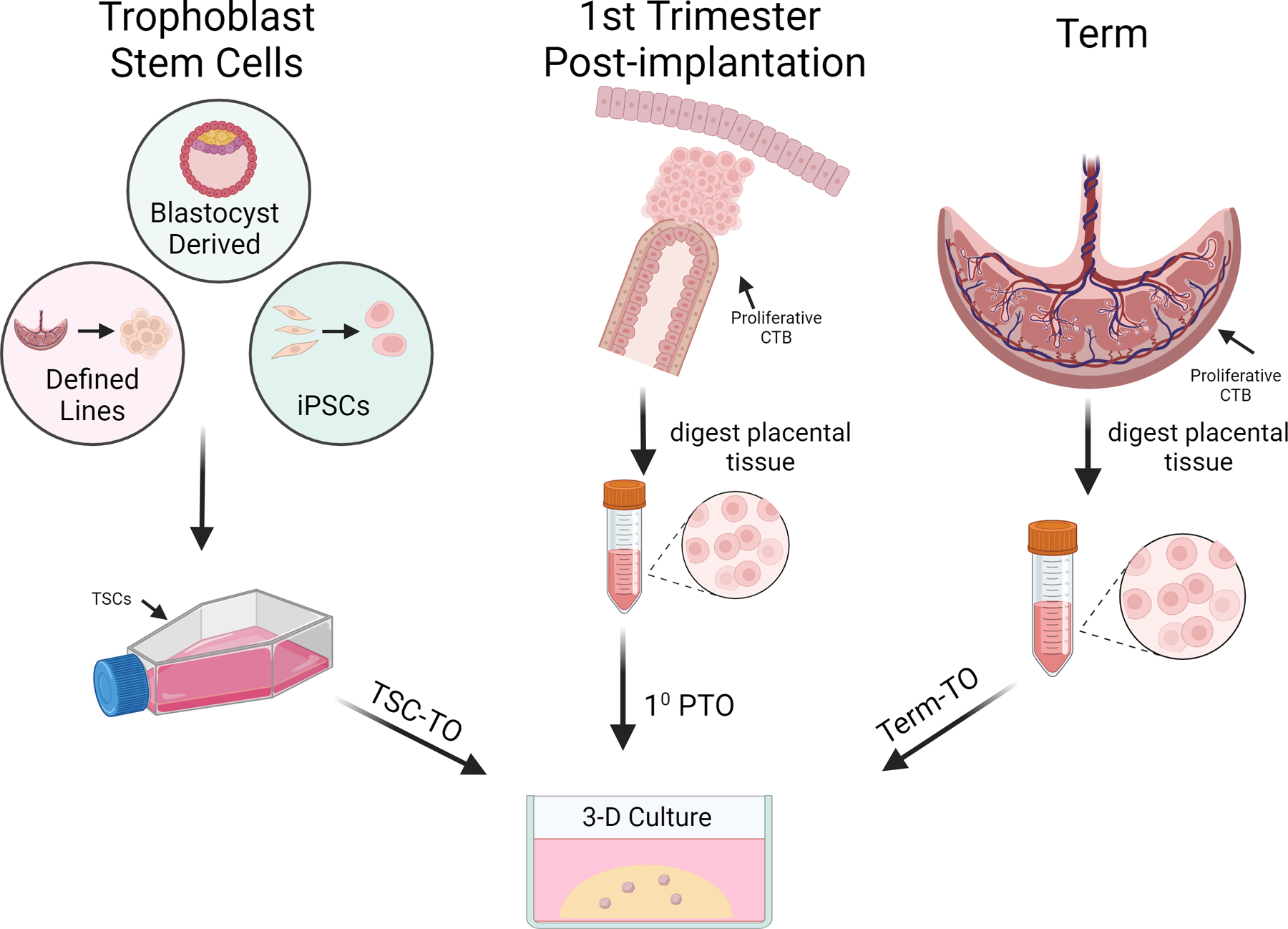
Overview of trophoblast organoid sources. Routes of access to trophoblast organoids. The trophoblast stem cell route has three starting points, all of which converge on TSC culture in 2D. Primary cell isolations from the placenta can be obtained from first trimester (middle) and term (right) samples. These tissues are both digested in a similar fashion to yield proliferative cytotrophoblast cells. All three routes converge when proliferative cytotrophoblast cells are placed into a 3D paradigm, yielding trophoblast organoids.

Trophoblast organoids can be derived through three principal routes: culturing primary trophoblast cells from first trimester samples (PTOs), culturing TSCs (TSC-TOs), or through isolation from term placental samples (illustrated in Figure 2) [10,48–50]. The variety of routes for establishing TOs in culture aids in building a robust model resource, but there are some notable differences in the behavior of TOs derived from different means, as illustrated in Figure 2. The structure of different TO models may vary, and this provides both diversity and adaptability to studies based on EVT or SYN dynamics. PTOs are appealing to researchers with access to first-trimester samples, and the protocols for derivation are adaptable and adequate for achieving high success rates prior to roughly 12 weeks of gestation. TSC-TOs are more widely accessible, and are amenable to many standard culture techniques, such as transfection, which is notoriously difficult to perform with primary tissue cultures. Taking transformed TSCs and placing them in the 3D paradigm allows for the study of genes suspected to be involved in trophoblast differentiation in a robust, representative in vitro environment. Term TOs (TTOs) present the possibility of being the most accessible to researchers with placental samples while simultaneously alleviating ethical concerns that surround samples from earlier pregnancy. While each route offers positive aspects, there are differences that will be discussed in this section, and leveraging certain aspects in the TO portfolio must be considered when designing a study.

### 3.2 Trophoblast Organoid Polarity

Anatomically, there are two main forms of TOs: inverted TOs and apical-out TOs. The primary features of inverted TOs are a proliferative CTB outer layer, surrounded by a core of SYNs [48–50]. This configuration is the opposite of how the villous placenta orientates in vivo. The exact mechanisms governing trophoblast polarity are not resolved, but the composition of basement membrane extracts/hydrogels Matrigel or Geltrex direct their polarity to an inside-out orientation. Thus, the inverted polarity is a result of the artificial culture environment. Working towards chemically defined hydrogels may allow for greater control over the experimental system. The inaccessibility of the SYN layer means that standard hydrogel culture is not as suitable for studies based on mechanisms in SYNs, but it is suitable for EVT-based studies. Alternative approaches have yielded results including the return of polarity to a physiological state, with the SYN layer on the outside of the organoid body [51–53]. After removal of basement membrane extract, apical-out TOs begin to morph into the SYN-out state over the course of several days, as shown by marker distribution and histology showing microvilli forming on the apical face of the organoids [51,52]. Evidence suggests that the orientation of the SYN layer also impacts the level of hormone, cytokine, and chemokine secretion, with apical-out TOs showing elevated SYN hormone secretion and varying immune profiles [51,52]. Several media manipulations may be performed to promote stronger SYN differentiation and ‘smoother’ organoids [51]. Incubating organoids in apical-out conditions for 48 h yields the consistent expression of SDC1 on the outer membranes of the organoids [53]. Indeed, it appears that control over the culture conditions with or without matrix hydrogels offers exquisite control over apical orientation, as few organoids cultured in suspension maintain their inside-out orientation, and reliably adopt the apical-out stance. Alternatively, the utilization of microbeads and seeding cultures within matrix hydrogels may offer a secondary route to achieving an appropriate orientation. This has been demonstrated in JEG-3 cells by utilizing feeder cells and encouraging attachment in 2D culture prior to transitioning to 3D [54]. Although the manipulation of orientation can be favorable in some applications, transitioning to suspension culture from hydrogel has proven to be terminal, and further passages are not sustainable, which impedes longer-term utility [52]. It has also been noted that apical-out organoids may not survive the duration of the EVT differentiation process [52]. The foundational work in TOs has been done on inverted TOs and will be discussed further, with apical-out models being a recent development. Both systems can capture the cellular dynamics of the first trimester, with inverted TOs being most suitable to EVT and CTB studies, while apical-out TOs are competent SYN models.

Primary TOs closely recapitulate the landscape of the first-trimester placenta. Transcriptomic studies have shown that PTO cells consistently and closely cluster with villous trophoblast tissues, and differentiated EVTs cluster with their in vivo counterparts [10,12,16]. Isolations performed on tissues younger than 12 weeks’ gestation are reportedly the most successful [48,49]. Indeed, staining for proliferation marker Ki67 shows stronger expression in these tissues, and the TSC niche in vivo appears diminished, but present, in later gestation [52]. Once established, PTOs can be maintained through at least 18 passages, with passage intervals of 7–10 days [55]. This is a notable improvement over previous 2D primary cell isolations, which were limited in passage number. However, this is not unique to TOs themselves, but to the establishment of the TSC niche in culture. PTOs also confer the advantage of modeling patient genetic backgrounds, which is important in a preclinical model. The mechanisms of how genetic changes on the maternal and fetal sides may predispose the behavior of PTOs is a growing area of research, with work done on recurrent pregnancy loss patients being a striking example of how PTOs can capture this aspect of development [56].

Comparing PTOs to TSC-TOs yields valuable insights into both the behavior of TSCs and the importance of a 3D culture paradigm to sustaining trophoblast identity. Evaluation of the comprehensive criteria for trophoblast identity proposed by Lee et al. in 2016 shows that both PTOs and TSC-TOs express appropriate markers for trophoblast identity, such as hypomethylation of the ELF5 promoter and cluster 19 miRNA expression [20]. However, an interesting difference can be detected among PTOs and TSC-TOs: the expression of HLA-A/B. HLA-A/B is not expressed by trophoblasts in vivo, but expression has been detected in TSCs by themselves and in TSC-TOs [9]. Interestingly, there appears to be an impact of 3D culture alone on TSCs. Their expression of HLA-A/B decreases when cultured in the 3D paradigm, suggesting that organoid cultures are more suitable for modeling trophoblast dynamics. Furthermore, TSC-TOs show limited fusion potential compared to PTOs, with fewer SYN lacunae detected inside the organoid bodies [9]. In fact, column-like cellular phenotypes are more prevalent in TSC-TOs as opposed to PTOs [8]. TSC-TOs much more closely recapture the CCC niche in the placenta, whereas PTOs capture the villous environment [8–10]. Both varieties can differentiate into EVT cells under the proper media conditions, but certain lines of TSC-TOs have shown differing capacities to achieve EVT phenotypes [21]. These factors must be considered when aiming to use TSC-TOs. Despite these subtle differences, TSC-TOs are still an excellent resource. Stem cell cultures are more amenable to gene knockouts and plasmid transfections than primary cells. Given that TSC-TOs do spontaneously differentiate into SYNs, even if at a lower rate, the dissection of genes regulating fusion can begin. Furthermore, TSC-TOs have proven to be readily adaptable to apical-out culture [51].

Term TOs have not been as comprehensively characterized, but the initial data suggest that they do capture trophoblast identity [52]. They are capable of modeling second- and third-trimester dynamics of infection and development and can be cultured in suspension-based methods to produce apical-out TOs [50]. The ability of TTOs to model early gestational phenotypes is yet to be investigated. They are highly proliferative and express appropriate markers for major trophoblast cell types. This is of critical importance due to the constrained access to early gestational samples. If culture media can direct the TSC niche into a first trimester-esque state, the value of TTOs increases dramatically due to their ability to not only model late gestation, but also a range of earlier gestational timepoints. The multiplicity of routes to obtain TOs has improved access for researchers to precious placental tissue, while it has simultaneously enabled intensive study on the mechanisms of trophoblast differentiation.

### 3.3 Trophoblast Cell Diversity Is Captured In Vitro by Trophoblast Organoids

The value of TOs as a model is determined by their ability to capture the cellular mechanics observed in vivo. To that end, scRNA-seq and flow cytometry studies performed on TOs of various origins have provided excellent insight [10,21]. While not all cell types seen in vivo can be accurately captured (namely giant cells), evidence suggests that many critical cell types can be adequately represented by TOs, and further work may unveil routes for further representations of the trophoblast clade. All types of TOs have their place in studying the mechanisms of trophoblast development. PTOs are unique because they can capture the genetic background of a patient and offer the possibility of abrogating diseased phenotypes—disentangling the contributions of the fetal trophoblasts and maternal decidua. TSC-TOs open the toolkit of molecular biology, being more amenable to techniques such as transfection to induce gene knockdowns or overexpression. Furthermore, the ability to induce TSCs or use naïve pluripotent stem cells makes access to this particular model amenable [10,11,45]. The appropriate expression of trophoblast identity genes bolsters the confidence that TSC-TOs can capture trophoblast diversity in vitro.

Primary TOs have been shown to possess more cellular diversity than their TSC-TO counterparts. The CTB niche is more conserved amongst PTO models, with EVT differentiation mechanics being more like what is seen in vivo. The progression of primitive EVTs to Notch1-positive progenitors with ITGA1 upregulation followed by that of ITGA5 as a mature phenotype is conserved more strongly in PTOs than TSC-TOs [8]. This is in line with previous work done in TSC-TOs, which showed a weaker expression of ITGA1 [10]. Further adding to this, analyses of key transcriptional factors have shown that PTOs are enriched as compared to TSC-TOs and are more representative of the placenta tissue [8]. However, it must be noted that TSC-TOs still distinctly capture the main developmental trajectories of trophoblasts, and it is possible that further media modifications and tailoring may reveal a more appropriate model, comparable to PTOs [8].

Taken together, however, the evidence that both varieties of TO can capture the main trophoblast cell states in both differentiation pathways is strong. Even in a less robust TSC-TO environment, the cell states of CTBs, TSCs, SYNs, fcCTBs (marked by ERVFRD-1), ccCTBs, and EVTs are clearly represented [8,10,21]. Leveraging the robustness of this model, themes around trophoblast differentiation and key signaling events governing the placenta have been uncovered. This highlights not only the utility of TOs moving forward but also their true power as an innovative technology in research.

A critical shortcoming of the model that must be addressed is the lack of other cell types. The first trimester placenta is mainly composed of trophoblast cells, but stromal cells, endovascular cells, and fetal immune cells (Hofbauer cells) all play considerable roles in placental development [4]. The development of co-culture organoid models that can capture the interplay between these other cell types and the trophoblast would improve the translational potential of this model, as well as uncover paracrine and endocrine signaling from the fetus that governs placental development.

### 3.4 Themes of Differentiation as Captured in Trophoblast Organoids

**Figure 3.**
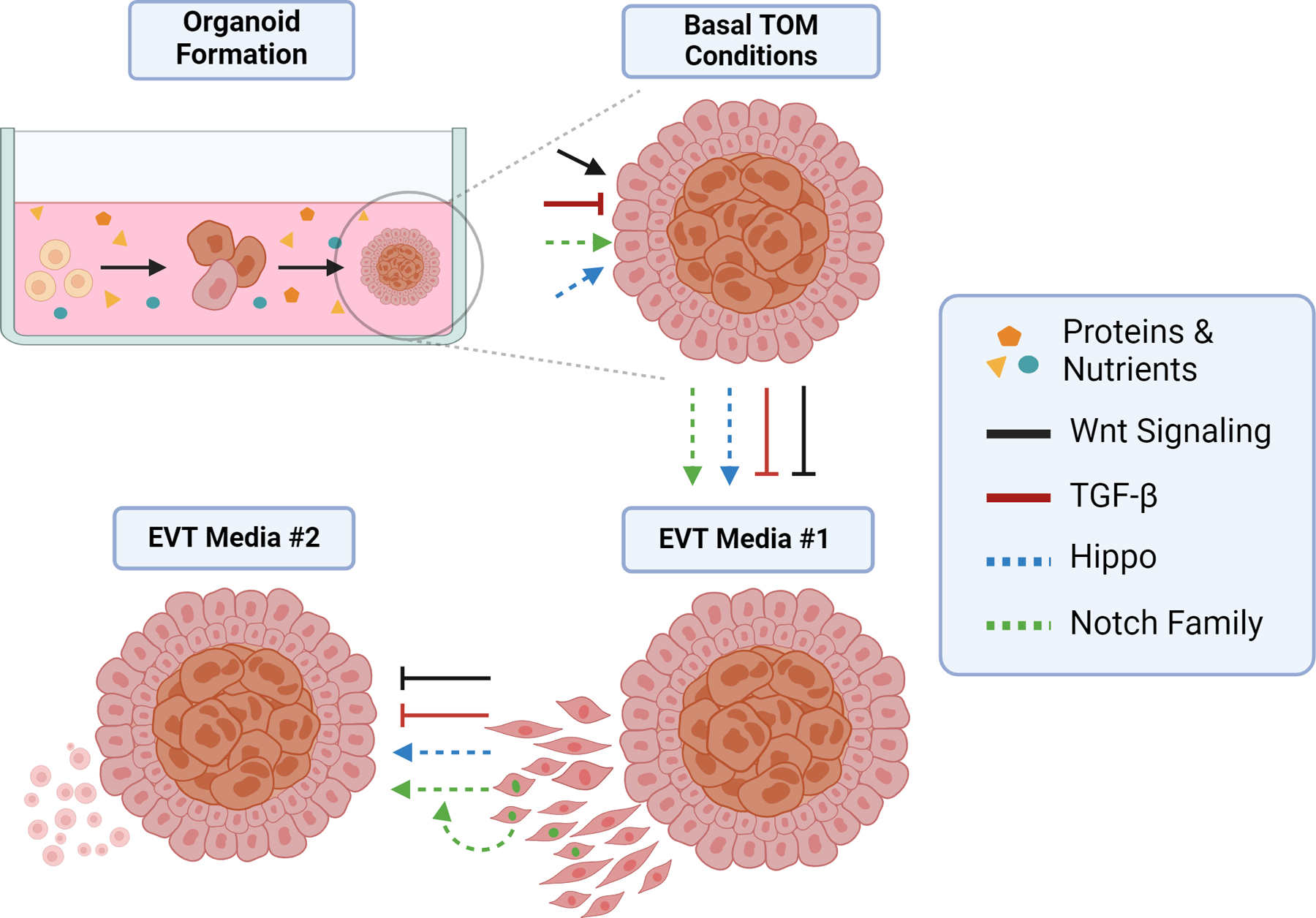
Signaling pathways in trophoblast organoids. A broad overview of key signaling pathways that are known to govern trophoblast differentiation in TOs. Media-manipulated pathways are shown with solid lines, whereas indirect/autocrine-activated signaling is shown with dashed lines. The contributions of TO media EVT#1 and EVT#2 (differing growth factor compositions) are shown with arrows. Inhibition of Wnt drives EVT differentiation. TGF-β is continuously inhibited in this model but may not always be.

With much ongoing work in the field surrounding trophoblast differentiation and development, several key themes have arisen in the derivation and establishment of TOs. First, there are key media manipulations that can control the preferred pathways of differentiation in PTOs, TSC-TOs, and TTOs. These manipulations have converged on the Wnt and TGF-β pathways. Secondary to this is the establishment of other key signaling pathways associated tightly with trophoblast identity. Namely, the Hippo and Notch pathways have been found to be strongly influential, and they can delineate cell states. This speaks to an intertwined field of regulation between autocrine and paracrine signaling in the placenta [49]. A comprehensive overview of these themes is necessary to tie together our understanding of the first trimester, and to identify areas ripe for experimentation and new knowledge. The roles of directly manipulated and indirectly manipulated pathways are shown in Figure 3.

## 4. Signaling Pathways in Trophoblast Organoids

Media manipulations are key to tight control in any experimental system, and TOs are no exception. The propensity of TOs to differentiate into the respective EVT or SYN cell types depends on experimental manipulation. While some self-induced differentiation to EVTs is reported in basal TO media, the main program conserved is the villous environment [8,9]. In this realm, the action of the Wnt and TGF-β pathways work in concert to govern trophoblast identity (Figure 3). The canonical Wnt pathway has been found to be a key regulator of stemness in the placenta, while negative TGF-β regulation is crucial to conserving the villous niche.

### 4.1 The Role of Wnt Signaling

The Wnt pathway is well known to those who study stem cells, and it is implicated in maintaining stem cell niches across many different tissues. The Wnt pathway can be divided into canonical and non-canonical classifications, which differ in whether β-catenin accumulates in the nucleus (canonical) or if the signal transduction occurs through other means (non-canonical) [57]. When β-catenin is stabilized in the cytoplasm, it is transported to the nucleus, where it associates with various TCFs (dependent on cellular context) to influence transcription. For the context of placental development, the focus will be on the canonical pathway. This does not preclude the involvement of non-canonical mechanisms; rather it implies that data obtained so far has focused on the demonstrable canonical impacts of Wnt signaling [58,59].

In trophoblast organoids, Wnt signaling is crucial for maintaining the TSC niche. The omission of CHIR99021, a chemical inhibitor of GSK3, which degrades β-catenin, results in non-proliferative organoids [48,49]. Furthermore, Wnt potentiator R-spondin, which serves to amplify the signal, aids in promoting the proliferation of TOs [48,49]. However, the loss of Wnt signaling does not lead to cell death. Rather, it has been found that the loss of Wnt activators leads to the spontaneous differentiation of TOs into invasive, EVT-like cells. These spindly cells appear within days of Wnt withdrawal. The expression patterns of TCFs 1–4 also serve to inform the activity of Wnt signaling in TOs and the placenta as a whole. Within the CTB compartment, TCF1-positive cells exist, although expression is not continuous across CTBs—it is possible that TCF1-negative cells represent an early step in the commitment to fcCTB/SYN formation. Notch1-positive ccCTBs did not express any TCF proteins, which is consistent with observations in vitro that the removal of Wnt leads to EVT development. Interestingly, TCFs 3/4 have been observed in later stages of EVT differentiation, both in vivo and in vitro [49]. This, coupled with the fact that EVT media lack Wnt ligands, suggests the possibility of autocrine Wnt activation occurring in ccCTBs as they differentiate into true EVTs—which is congruent with evidence from 2D culturing of EVTs [60]. Overall, there is evidence that TO models are capable of recapitulating Wnt signaling complexities as observed in vivo.

### 4.2 The Role of TGF-β Signaling

In contrast to the stem regulation provided by the Wnt family, the TGF-β family has been shown to control the invasive potential of trophoblast cells [61]. The TGF-β superfamily of proteins consists of numerous ligands and receptors, including ligands of TGF-βs, GDFs, and BMPs, and nodal ligands, whereas a roster of receptors including ALKs 1–5 and TGF-βRs exist to sift this diversity. Members of the TGF-β superfamily have well-established roles in pregnancy, and all three main varieties of trophoblast cells display rich expression of both ligands and receptors. The maternal decidua includes many TGF-β members too [61]. Undoubtedly, the emergence of TOs and TSCs has set the stage for finer investigations of these components. Already, new data have emerged regarding the role of the ALK-5 receptor and TGF-β1 ligand.

TGF-β1 has emerged as a crucial regulator of the transition from trophoblast stem cells/CTBs into EVTs. In TO media, inhibition of the TGF-β pathway is achieved via A83–01, a chemical inhibitor of ALK5. By inhibiting ALK5, the signal transduction is completely blocked with no phosphorylation of SMAD3. Indeed, even exogenous supplementation of TGF-β cannot overcome inhibition through ALK5 done by A83–01. The action of TGF-β has been revealed via withdrawing it from culture conditions. Initially, EVT differentiation is sparked by the withdrawal of Wnt and Wnt promoting factors (CHIR99021 and R-spondin 1). A constant inhibition of TGF-β remains in many EVT protocols as of this writing, yet evidence suggests that this may be a hindrance. EVTs in vivo express not only HLA-G, but also other markers specifically associated with more mature phenotypes [61]. DAO and PAPPA are key markers expressed by mature EVTs, namely iEVTs, found within the decidua. Differences in the expression of markers were underlined by a change in morphology [61].

The morphology of in vivo EVTs differs from current in vitro-generated EVTs, which have a rounder, less spindle-like shape. By sequentially withdrawing Wnt, followed in roughly 5 days by withdrawing A83–01, and, thus, activating TGF-β, trophoblast organoids demonstrated more robust EVT phenotypes, with autocrine expression allowing for DAO and PAPPA to be detected [61]. Adding in exogenous TGF-β yields an even stronger expression of these markers. The morphology of these EVTs differs from TGF-B-inhibited EVTs, which adopt a rounder shape reminiscent of in vivo EVTs. The phosphorylation of SMAD3 is a critical effector of this mechanism, as evidenced by A83–01. Autocrine activation of TGF-β receptors does not abolish the inhibition of SMAD3; thus, constant inhibition does not allow for autocrine action for the self-regulation of the phenotype.

### 4.3 The Hippo Pathway

Moving on from direct media manipulations, observations in the placenta itself and within TOs have suggested significant roles for both Hippo and Notch signaling. Direct actors in the Hippo pathway are difficult to resolve, as it can respond to more obscure signals such as extracellular matrix rigidity. However, clear expression patterns for key transcriptional factors YAP and TAZ, and, ultimately, TEAD4, suggest that Hippo is essential to the proliferative capacity of CTB [62]. Notch signaling, on the other hand, is more diverse: there appear to be roles for three Notch family members, each associated with their own cell states.

The Hippo signaling pathway, whose first components were discovered in 1995, has critical roles in regulating stem cell renewal and proliferation and, in turn, governs organ size [63,64]. Briefly, the pathway operates through a kinase cascade that converges on transcriptional coactivators YAP and TAZ (WWTR1), which bind to TEAD proteins 1–4 to control the expression of stemness-associated genes. In the ‘off’ state, YAP/TAZ are present in the nucleus, where they are bound to a TEAD protein and, thus, the gene expression is active. In the ‘on’ state, YAP/TAZ are degraded in the cytoplasm, and nuclear accumulation is lost [64]. Without it, TEAD proteins cannot activate transcription.

In the context of the placenta, Hippo has been shown to operate through its transcriptional cofactors, YAP, TAZ, and TEAD4 to govern trophoblast stemness, although YAP and TAZ do not have equivalent roles, as is often described [56,65,66]. TEAD4 has emerged as a reliable marker of trophoblast stem cells, and its loss has been shown to hamper TSC proliferation and dramatically reduce the formation efficacy of TSC-TOs [56]. The isolation of TSCs from recurrent pregnancy loss placentae showed that TEAD4 expression was reduced, and organoid formation was significantly hampered. However, rescuing TEAD4 expression restored the capacity of these cells to form healthy organoids, at comparable sizes to those of healthy controls [56]. This study highlights the advantage of culturing patient-derived TSCs and using those to form TSC-TOs, while preserving the unique genetic background of a disease.

The distributions of YAP and TAZ have been shown to be different with respect to CTB and EVT identities. An inverse relationship has been observed in vivo based on microdissections, showing that CTBs express high levels of YAP, whereas EVTs express high levels of TAZ. TEAD4 expression is lost through differentiation, suggesting that YAP is a critical regulator in maintaining trophoblast stemness [65]. Nuclear YAP suppresses fusion genes, preventing SYN formation. Chemical inhibition of YAP in PTOs led to the loss of CTB cells [65]. Following up on the observations about TAZ, it is intriguing to note that the loss of TAZ results in the loss of the proliferative capacity of TSC-TOs, and they cannot be maintained through one passage [66]. Furthermore, the depletion of TAZ leads to SYN induction and a loss of EVT phenotypes. This strongly suggests that TAZ is critical in promoting EVT differentiation whilst preventing inappropriate SYN development [66]. Taken together, this evidence suggests the Hippo pathway is responsible for governing CTB stemness and preventing precocious SYN differentiation, while in the column, it also fosters EVT development. Crosstalk between this signaling pathway and the Wnt pathway has been observed, adding a layer of complexity to the picture [66]. Delineating the nature of Hippo/Wnt crosstalk may play a critical role in understanding EVT differentiation and how EVTs are able to integrate spatial and environmental cues to appropriately regulate their invasive depth.

### 4.4 Notch Family Signaling

The Notch signaling pathway features highly conserved elements regulating cell fate, proliferation, and cell death, to name a few. The basic paradigm of Notch signaling includes the ligand binding of Delta-like ligands or Jagged ligands to the Notch receptor. Ligand binding triggers the proteolytic cleavage of the Notch intracellular domain (ICD), which translocates to the nucleus as the effector unit of the signal [67]. In the placenta, there appear to be diverse roles for the various Notch receptors [68]. Notch1 has been established as a distinct marker for ccCTBs, the first step along the EVT pathway [13]. The roles of Notch2 and Notch3 have also been investigated, while Notch4 has been shown to be present in first-trimester CTBs. Thus, every component plays a role, depending on the cellular context.

Trophoblast organoids exhibit similar Notch1 expression patterns, as observed in vivo. Shortly after the deprivation of Wnt factors and removal of TFG-B inhibition, Notch1-positive cells develop on the outer edges of TOs, in a manner that is highly comparable to that of ccCTB cells [49]. Furthermore, these cells are not yet HLA-G-positive, and they instead capture the transitional state of the column. Notch1 actively represses the stemness of trophoblast cells in the column, pushing them towards the EVT phenotype [13]. Notch3, however, is expressed in CTBs, where it may play a critical role in promoting these cells’ proliferative capacity. Indeed, PTOs exposed to inhibitory protein DN-MAML1 show elevated cell fusion and SYN markers. Conversely, TSCs transfected to overexpress Notch3-ICD experienced an increase in proliferation, but the behavior of TOs in this paradigm is unknown. Intriguingly, Notch3’s presence is noted at the tips of anchoring villi, in the ccCTB niche, suggesting further roles for Notch3 in regulating the trophoblast cell fate [69]. Notch2 localizes to HLA-G-positive EVTs alongside distal ccCTBs, suggesting a switch between Notch1- and Notch2-positive cells. Notch2 has been shown to inhibit EVT migration, but inhibition had no impact on cell proliferation [70]. It is worth noting that Notch4 has been shown to localize to CTBs, but the exact role it plays and its presence in TO model systems has not been confirmed [71]. While much has yet to be defined for the specific contexts in which the members of the Notch family orchestrate the proliferation and differentiation of cytotrophoblasts, TOs can capture important subtleties needed to effectively study these events, particularly in a combinatory approach with other in vitro models.

## 5. Translational Potential

The ability of TOs to capture the first trimester (and perhaps all of gestation) has stationed them at the forefront of placenta research. Already, an intriguing usage has shown the potential for TOs to model not only normal pregnancy events, but also pathological ones [72]. Utilizing chorionic villous sampling (CVS), the possibility to capture PTOs from ongoing pregnancies makes for a powerful tool. CVS is a medical procedure in which ultrasound-guided needle placental biopsies can be obtained. Importantly, the procedure is not terminal, as is the case with obtaining standard placenta samples. CVS carries relatively minimal risk to the mother and fetus, with pregnancy loss in the 14 days following CVS occurring in 0.7% of transabdominal procedures [73]. As these biopsies consist of villous tissue, establishing organoids from this tissue offers a way to produce an in vitro snapshot of ongoing pregnancies. Crucially, since the procedure is not terminal, pregnancy outcomes can be considered in the analysis of frozen biobanked tissue. Indeed, this allows for pregnancy phenotypes to be captured in vivo alongside in vitro systems, and the extensive interrogation of diseased phenotypes may give clues to what genetic mechanisms are at play in gestational diseases.

For example, if early gestational organoids are established from a pregnancy that then becomes pre-eclamptic, this offers an opportunity to observe and experiment upon pre-eclamptic phenotypes in a way that is unrealized by current models. Indeed, reports already exist in the human literature of the successful use of CVS to generate organoids from pregnancies diagnosed with Down syndrome, a unique opportunity to study placental development in the context of genetic disease [72]. While many placental diseases are suspected to arise due to insufficient invasion and vascular remodeling, there have been no causative genes or mechanisms singled out. CVS-generated organoids offer a snapshot into current placental development at the individual level, and the detection of abnormalities in early gestation remains critical to protecting the health of the mother and unborn child. Access to diseased phenotypes that are stable in culture and genetically unmodified may allow for the testing of gene therapies and drugs for pre-eclampsia and other trophoblastic diseases.

CVS does come with a caveat: placental mosaicism has been found to be increasingly common, and a bottleneck effect of a small founding population of cells in organoid culture may cause them to not be truly representative of the placenta [74]. However, the extent of mosaicism and the impact it has, if any, on placental function, is subject to debate. Further studies are needed, and it is not the aim of this review to dissect such topics.

The potency of the CVS method has been demonstrated by Schaffers and colleagues. Using CVS, TOs were successfully generated from both healthy control patients, and one line each from a patient with Down syndrome (trisomy 21) and a patient with Cornelia de Lange syndrome (CdLs). The successful derivation of TOs occurred up to 14 weeks of gestation, but later attempts were unsuccessful. scRNA-seq experiments performed upon both control and diseased organoids revealed areas of overlap but, crucially, some significant divergence. The CdLs-TOs showed enrichment for senescence-associated transcripts as compared to standard TOs. Furthermore, these organoids were lacking in SYN cells, which formed regularly in control TOs [72]. This tracks with data about CdLs from both mice and humans and serves to demonstrate the translational potential of TO model systems in combination with CVS. Down syndrome TOs were not qualitatively different from their control counterparts. While this study has no statistical power (*n* = 1 for both diseases), it does show proof of concept and lays a foundation for the further study of gestational/developmental diseases that are believed to be trophoblastic in origin in ongoing pregnancies.

The development and refinement of CVS protocols are also ongoing in studies using NHPs [75]. The successful derivation of CVS-based TOs in the NHP can save crucial resources, as primate pregnancies are resource-intensive, and animal shortages can be a limiting factor in all research settings. Furthermore, the opportunity to study experimentally manipulated pregnancies in parallel in vivo and in vitro will yield new insights into placental development and pregnancy diseases. Established primate models for chronic Western-style diets, exposure to alcohol, and Zika virus infection are well characterized, allowing for extensive validation to show that in vitro models accurately recreate these phenotypes [76–78]. The impacts of these broad lifestyle and environmental factors on the placenta are often unknown or only examined by observation, without the rigor of experimental data. The translational value of this knowledge cannot be understated, as any experimental manipulation during pregnancy is prohibited. Yet, these conditions are observed clinically and studying them in this manner could generate treatments and insights for pregnancy health. Using NHPs to further validate TOs as a model for human clinical research underscores the necessity of primate models and the critical gap they bridge in translational research.

## 6. Future Directions and the Role of NHP Research

While CVS-based organoids represent an exciting avenue for the field, it is not the only route to be explored. Many questions remain about the role of the low-oxygen environment in the first trimester, and, in that regard, how it may influence trophoblast cells. There are conflicting reports on the role of hypoxia-inducible factor in early placenta development, but many of these studies have relied on alternative cell lines rather than the robustly defined trophoblasts used in TO systems [79,80]. This may become a shortfall when results are extrapolated to the in vivo setting. Furthermore, the refinement of differentiation protocols is still ongoing, with the role of TGF-β being highlighted and the capturing of deeply invasive, mature EVTs, namely iEVTs and eEVTs, in the TO model paradigm being established.

The NHP offers an excellent resource to bridge translational gaps in humans, leveraging both in vitro and in vivo aspects at the same time. Simply put, in vivo pregnancy manipulation cannot be achieved in humans. This has caused us to rely on translational animal models, and developing organoid models alongside them may aid in not only validating the translational efficacy of organoids, but in animal welfare and the minimization of animal use in research. This is an area of active research, with the very recent establishment of mTSCs in culture being a huge milestone for NHP-based models. Once fully established and characterized, NHP trophoblast organoids have the potential to provide a platform to perform mechanistic studies of adverse stimuli that are implicated in pathological pregnancy outcomes and toxicity studies, and for the pre-clinical testing of therapeutics for use in pregnancy.

Finally, co-culture methodologies utilizing both maternal and fetal tissue have been suggested. Decidual organoids that are hormone-responsive and capture the maternal side of the placental interface have been developed, and they display robust proliferation and are readily isolated [81]. Their influence on TOs has been minimally explored, but the decidua is known to secrete many growth factors and hormones that regulate trophoblast expansion and invasion [61]. Exploring the maternal aspects of trophoblast invasion and differentiation will further our understanding of the placenta while also offering an added level of complexity in culture systems capturing tissue from both parts of the maternal/fetal interface.

In summary, trophoblast organoid models offer unprecedented access to the first trimester and may be able to model all phases of gestation. The recent expansion of knowledge around the cell dynamics of the first trimester has uncovered new cell types, and these can be represented in vitro. The accessibility of TOs is greatly improved by TSC-TOs, but PTOs are the most representative model at present, while TTOs offer the possibility to model late gestation. The NHP has also seen an expansion with the isolation of mTSCs, and the translational ability of this model must not be overlooked. Deeper studies with TOs have revealed key themes around the maintenance and expansion of the key trophoblast cell states: the signaling networks of CTBs, SYNs, and EVTs are beginning to be resolved. The advent of CVS-derived TOs ushers in an exciting possibility of capturing snapshots of pregnancy and increasing our knowledge of pathological phenotypes. The future promises to bring clarity and resolution to the mechanisms that underly trophoblast development and differentiation, which will benefit human health.

## Figures and Tables

**Table 1.** Trophoblast cell subtype-specific markers.

Cell Type	Human	Rhesus Macaque
Trophoblast	KRT7	KRT7
CTB	GATA3, TP63, TEAD4, CDH1	KI67, TEAD4
fcCTB	ERVFRD-1	ERVFRD-1
ccCTB	Notch1, KI67, ITGA1/2	Notch1, KI67
SYN	GCM1, SDC1	SDC1, Mamu-AG
EVT	HLA-G, ITGA5	NCAM1, ITGA5 Mamu-AG
iEVT	PAPPA, DAO, SERPINE1	N/A
eEVT	NCAM1	N/A
GC	CD81, RAC11	Unknown

## Data Availability

Not applicable.

## Abbreviations

CTB: Cytotrophoblast
SYN: Syncytiotrophoblast
EVT: Extravillous Trophoblast
NHP: Nonhuman Primate
TO: Trophoblast Organoid
TSC: Trophoblast Stem Cell
fcCTB: Fusion-Competent Cytotrophoblast
CCC: Cytotrophoblast Cell Column
iEVT: Interstitial Extravillous Trophoblast
eEVT: Endovascular Extravillous Trophoblast
mTSC: Macaque Trophoblast Stem Cell
mCG: Macaque Chorionic Gonadotrophin
hCG: Human Chorionic Gonadotrophin
PTO: Primary Trophoblast Organoid
TSC-TO: Trophoblast Stem Cell Trophoblast Organoid
TTO: Term Trophoblast Organoid
GC: Giant Cell
ICD: Intracellular Domain
CVS: Chorionic Villous Sampling
CdLs: Cornelia de Lange Syndrome
